# Low-Intensity Vibration Improves Angiogenesis and Wound Healing in Diabetic Mice

**DOI:** 10.1371/journal.pone.0091355

**Published:** 2014-03-11

**Authors:** Eileen M. Weinheimer-Haus, Stefan Judex, William J. Ennis, Timothy J. Koh

**Affiliations:** 1 Department of Kinesiology and Nutrition, University of Illinois at Chicago, Chicago, Illinois, United States of America; 2 Center for Tissue Repair and Regeneration, University of Illinois at Chicago, Chicago, Illinois, United States of America; 3 Department of Surgery, University of Illinois at Chicago, Chicago, Illinois, United States of America; 4 Department of Biomedical Engineering, Stony Brook University, Stony Brook, New York, United States of America; Medical College of Georgia, United States of America

## Abstract

Chronic wounds represent a significant health problem, especially in diabetic patients. In the current study, we investigated a novel therapeutic approach to wound healing – whole body low-intensity vibration (LIV). LIV is anabolic for bone, by stimulating the release of growth factors, and modulating stem cell proliferation and differentiation. We hypothesized that LIV improves the delayed wound healing in diabetic mice by promoting a pro-healing wound environment. Diabetic db/db mice received excisional cutaneous wounds and were subjected to LIV (0.4 g at 45 Hz) for 30 min/d or a non-vibrated sham treatment (controls). Wound tissue was collected at 7 and 15 d post-wounding and wound healing, angiogenesis, growth factor levels and wound cell phenotypes were assessed. LIV increased angiogenesis and granulation tissue formation at day 7, and accelerated wound closure and re-epithelialization over days 7 and 15. LIV also reduced neutrophil accumulation and increased macrophage accumulation. In addition, LIV increased expression of pro-healing growth factors and chemokines (insulin-like growth factor-1, vascular endothelial growth factor and monocyte chemotactic protein-1) in wounds. Despite no evidence of a change in the phenotype of CD11b+ macrophages in wounds, LIV resulted in trends towards a less inflammatory phenotype in the CD11b− cells. Our findings indicate that LIV may exert beneficial effects on wound healing by enhancing angiogenesis and granulation tissue formation, and these changes are associated with increases in pro-angiogenic growth factors.

## Introduction

Chronic wounds represent a significant health problem, especially in diabetic patients. Of the 150 million people with diabetes worldwide, the lifetime incidence of a foot ulcer is ∼25% and up to 70% of these wounds remain unhealed after 20 weeks of standard treatment [1], [2]. Normal wound healing consists of overlapping phases of inflammation, tissue formation and angiogenesis, and remodeling, but in chronic wounds associated with diabetes all of these phases are compromised [2]. A prolonged accumulation of inflammatory cells, elevated levels of inflammatory cytokines and reduced levels of pro-angiogenic and pro-healing growth factors contribute to the impaired angiogenesis and healing [2].

Mechanical energy-based modalities (i.e. negative pressure wound therapy, ultrasound) are often used in conjunction with standard treatments for chronic wounds. These treatments use local mechanical stimulation of the wound in an attempt to modify the cellular and biochemical environment to improve angiogenesis and healing [3]. Currently, there is much debate over the efficacy of these treatments and the quality of evidence is often questioned. While there is some evidence that these therapies may improve wound healing, a lack of larger controlled studies of high methodological quality has amounted to insufficient evidence in proving an additional benefit [4], [5].

In the current study, we investigate a novel therapeutic approach to wound healing – whole body low-intensity vibration (LIV). LIV has been extensively studied in humans to promote musculoskeletal anabolism [6], [7], [8], although the precise mechanisms that modulate these cellular responses are unclear. During LIV, subjects stand on a platform that delivers uniform vertical oscillations at various amplitudes (∼0.2–0.4 g) and frequencies (∼30–90 Hz). Daily exposure to LIV for 10–20 minutes has provided promising results as a non-invasive treatment for osteoporosis by increasing production of growth factors, modulating stem cell proliferation and differentiation, and increasing bone mass in individuals with low bone mineral density [9], [10], [11], [12], [13]. Furthermore, a single bout of LIV has been shown to increase systemic and regional (i.e. skin) blood flow [14], [15], [16], [17]. Thus, although LIV has not yet been investigated as a means to promote skin wound healing it has the potential to induce mechanotransduction both locally in the wound as well as at distant sites that results in a more pro-healing biochemical environment of the wound as well as improved tissue perfusion.

The purpose of this study was to assess the effects of whole-body LIV on wound healing in diabetic db/db mice. The central hypothesis of this study was that LIV improves the delayed wound healing in diabetic mice by promoting a pro-angiogenic and pro-healing wound environment.

## Methods

### Animals

Diabetic db/db mice, which are widely used as a model of delayed healing, were obtained from Jackson Laboratories. Experiments were performed on male mice 12–16 week-old. Only mice with fasting blood glucose >250 mg/dl were used. All procedures involving animals were approved by the Animal Care Committee at the University of Illinois at Chicago (protocol #12–207). All animals were housed under standard conditions and treated according to the Guide for the Care and Use of Laboratory Animals of the NIH.

### Excisional wounding

Mice were subjected to excisional wounding as described previously [18]. Briefly, mice were anesthetized with isoflurane and their dorsum was shaved and cleaned with alcohol. Four 8 mm wounds were made on the back of each mouse with a dermal biopsy punch and covered with Tegaderm (3 M, Minneapolis, MN) to keep the wounds moist and maintain consistency with treatment of human wounds. Wounds were harvested at 7 and 15 days post-wounding.

### Low-intensity vibration

**Figure 1:**
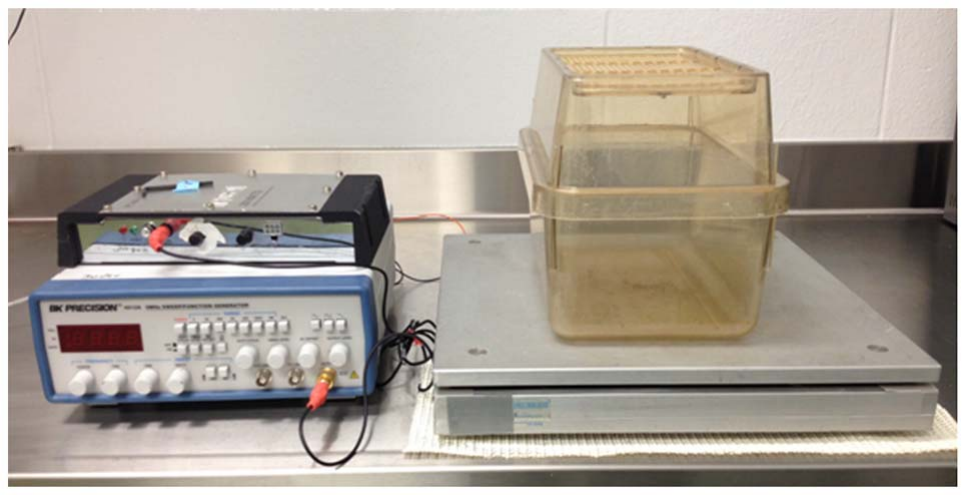
Image of low-intensity vibration (LIV) plate used for LIV treatments. Mice were placed in an empty cage directly on the vibrating plate, and LIV was applied vertically at 45 *g* for 30 min/d for 5 d/wk. The non-vibrated sham controls were similarly placed in a separate empty cage but were not subjected to LIV.

Following wounding, mice were randomly assigned to the LIV or a non-vibration sham (control) treatment. For the LIV, mice were placed in an empty cage directly on the vibrating plate, and LIV was applied vertically at 45 Hz with peak acceleration of 0.4 *g* for 30 min per day for 5 days/week starting on the day of wounding (Figure 1). The non-vibrated sham controls were similarly placed in a separate empty cage but were not subjected to LIV. The mechanical signals were calibrated using an accelerometer attached to the inside surface of the bottom of the cage, so that the signals produced were indeed those transmitted to the feet of the mice. In addition, the amplitude of the vibrations (0.4 *g*; ∼30 µm displacement) are small enough that the cage does not move relative to the plate and the vibrations of the plate and the cage are in sync.

### Wound healing assays

Wound healing was assessed histologically using our previously published assays of re-epithelialization, granulation tissue formation, collagen deposition, and angiogenesis using hematoxylin and eosin, Masson's Trichrome and CD31 stained cryosections [18], [19]. For all wound healing analyses, digital images were obtained using a Nikon Instruments Eclipse 80i microscope with a 20×/0.75 objective, a DS-Fi1 digital camera, and NIS Elements software.

#### Tissue preparation

Two wounds per mouse were collected and sectioned from one edge to well past the center. Sections were then selected from the center of the wound by microscopic assessment. Three 10-µm sections judged to be at the actual center of the wound were used for re-epithelialization and granulation tissue thickness measurements. Adjacent three 10-µm sections were used for trichrome staining, CD31 staining, and inflammatory cell staining.

#### Re-epithelialization and granulation tissue thickness

Wound re-epithelialization was measured by morphometric analysis of wound sections. Sections taken from the center of the wound were stained with H&E. The distance between the wound edges, defined by the distance between the first hair follicle encountered at each end of the wound, and the distance that the epithelium had traversed into the wound, were measured using image analysis software. The percentage of re-epithelialization [(distance traversed by epithelium)/(distance between wound edges) ×100] and granulation tissue thickness [(area of granulation tissue present)/(distance between wound edges)] was calculated for three sections per wound and was averaged over sections to provide a representative value for each wound.

#### Collagen Deposition and Angiogenesis

Dermal healing was assessed using Masson's trichrome stain for collagen deposition and immunohistochemical staining for platelet-derived endothelial cell adhesion molecule-1 (also called CD31) for angiogenesis. For trichrome analysis, staining was performed according to the manufacturer's directions (IMEB, San Marcos, CA, USA), and image analysis software (Scion Image, Scion, Frederick, MD, USA) was used to quantify the percentage of blue collagen-stained area relative to the total area of the wound bed. For angiogenesis, an antibody against CD31 (BD Pharmingen, San Diego, CA, USA) was used in conjunction with procedures identical to those for inflammatory cells described below, and image analysis software was used to quantify the percentage of CD31-stained area relative to the total area of the wound bed. For each assay, digital images covering the majority of the wound bed (usually three images at ×20 magnification) were first obtained. The percent area stained in each image was then quantified by counting the number of pixels staining above a threshold intensity and normalizing to the total number of pixels. Threshold intensity was set such that only clearly stained pixels were counted. The software allowed the observer to exclude staining identified as artifact, large vessels, and areas deemed to be outside the wound bed. For both trichrome and CD31 staining, three sections per wound were analyzed, and data were averaged over sections to provide a representative value for each wound.

### Inflammatory Cell Accumulation

Immunohistochemical analysis was performed on cryosections. Sections were air-dried, fixed in cold acetone, washed with PBS, quenched with 0.3% hydrogen peroxide, and washed with PBS. Sections were blocked with buffer containing 3% bovine serum albumin and then incubated with F4/80 antibody to label macrophages (1∶100, eBioscience, San Diego, CA) or Ly6G antibody to label neutrophils (1∶100, BD Pharmingen, San Diego, CA). Sections were then washed with PBS and incubated with biotinylated anti-rat secondary antibody (1∶200, Vector Laboratories, Burlingame, CA). After a wash with PBS, sections were incubated with avidin D-horseradish peroxidase (1∶1000) and developed with a 3-amino-9-ethylcarbazole kit (Vector Laboratories). Digital images were obtained using a Nikon Instruments Eclipse 80i microscope with a 20×/0.75 objective, a DS-Fi1 digital camera, and NIS Elements software and the percent area stained in each image was then quantified as described above for trichrome and CD31.

### ELISA

Wounds were homogenized in cold PBS (10 ml of PBS per mg wound tissue) supplemented with protease inhibitor cocktail (Sigma Aldrich, St. Louis, MO, USA) using a dounce homogenizer and then centrifuged. Supernatants were used for enzyme-linked immunoassay of IL-1β, MCP-1, IL-10, TGF-β1 (eBioscience, San Diego, CA, USA) and VEGF and IGF-1 (R&D Systems, Minneapolis, MN, USA).

### Cell isolation

Cells were dissociated from excisional wounds using an enzymatic digest with collagenase I, collagenase XI, and hyaluronidase [18]. Neutrophils, T cells, and B cells were marked for depletion by incubating cells for 15 min with fluorescein isothiocyanate (FITC)-conjugated anti-Ly6G (1A8), anti-CD3 (17A2), and anti-CD19 (6D5) (1∶10; Biolegend); these cells were depleted from the total cell population using anti-FITC magnetic beads and by following the manufacturer's instructions (Miltenyi Biotec). Cells of the monocyte/macrophage lineage were then isolated using CD11b magnetic beads. Both CD11b+ and CD11b− cell fractions were collected and then stored at −80°C for later RNA analysis. Previous studies indicated that >90% of the CD11b+ cells thus isolated were positive for monocyte/macrophage markers Ly6C and/or F4/80 by flow cytometry [18]. The CD11b− cell fraction likely consists mainly of fibroblasts, endothelial cells and keratinocytes.

### RNA analysis

Total RNA was isolated from cells using the RNeasy kit (Qiagen). cDNA was synthesized using the Thermoscript RT-PCR System (Invitrogen). Real-time PCR was performed in a 7500Fast System (Applied Biosystems) using TaqMan Universal PCR Master Mix and TaqMan Gene Expression Assay primer/probe sets (Applied Biosystems). Relative gene expression was determined using the 2^−ΔΔCT^ method, and GAPDH served as the endogenous control gene.

### Statistics

Values are reported as means ± standard error. Measurements of wound healing were compared using a two-way (treatment and time) ANOVA. The Holm-Sidak post hoc test was used when ANOVAs demonstrated significance. Measurements of gene expression and protein levels (ELISA) were compared between treatment groups using a t-test (7 d time point only). Differences between groups were considered significant if *P*≤0.05.

## Results

### LIV promotes wound healing by increasing granulation tissue formation

**Figure 2:**
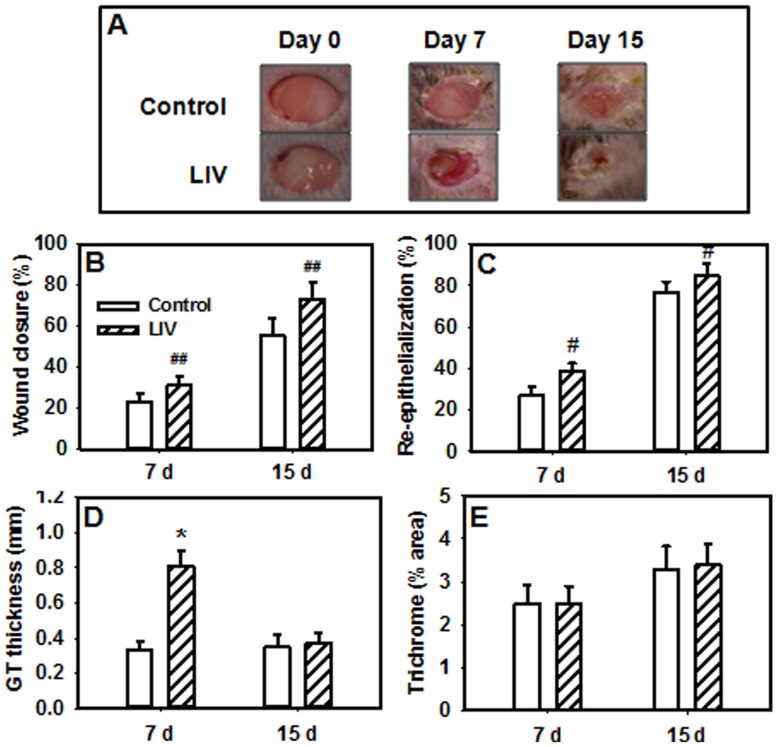
Low-intensity vibration enhances wound healing in db/db mice. Mice received either low-intensity vibration (LIV; 0.4 *g* at 45 Hz) or a sham control treatment starting the day of wounding for 5 d/wk. Representative images of wounds were taken on days 0, 7 and 15 post-injury (a). Wound closure was measured in digital images of wound surface, 7 d, n = 12–13 per group; 15 d, n = 3 per group (b). Re-epithelialization was measured in hematoxylin and eosin stained sections of wound center, 7 d, n = 13–16 per group; 15 d, 8–9 per group (c). Granulation tissue thickness was measured as the area of granulation tissue divided by the distance between wound edges, 7 d, n = 13–16 per group; 15 d, 8–9 per group (d). Collagen deposition was measured in trichrome stained sections as the percent area stained blue, 7 d, n = 13–16 per group; 15 d, 8–9 per group (e). ^#^Main effect of treatment, *P*≤0.05. ^##^Main effect of treatment, *P* = 0.066. *Mean value significantly different from that of control for same time point, *P*≤0.05.

Surface measurements revealed a trend towards accelerated closure in wounds from LIV-treated mice (main effect of treatment, P = 0.066) compared to wounds from control mice (Figure 2a, b). By day 15, wounds from LIV-treated mice exhibited closure of 73±9% the original wound size while wounds from control mice exhibited closure of only 55±9% of the original wound size. Histological measurements indicated that LIV increased re-epithelialization and granulation tissue formation on day 7 (Figure 2c, d), but effects on collagen deposition did not reach statistical significance (Figure 2e). Although the percent collagen staining in wounds was not different between LIV and control treatments on day 7, the increased amount of granulation tissue at this time point in the LIV-treated mice implies an increase in total collagen deposition. LIV-induced changes in wound healing were not mediated by changes in blood glucose levels (control, 427±25 mg/dL; LIV, 440±25 mg/dL).

### LIV increased angiogenesis and altered inflammatory cell accumulation in the wound

**Figure 3:**
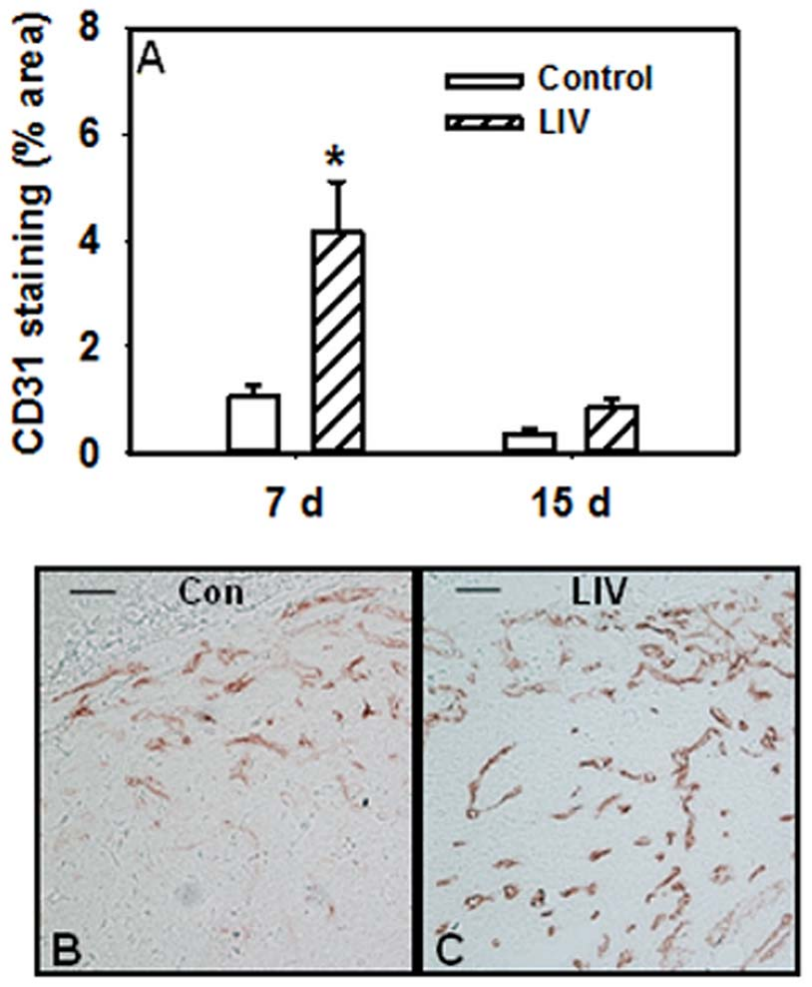
Angiogenesis is enhanced following low-intensity vibration. Wound sections were stained with antibodies against CD31 (a). Representative images of CD31 stained sections (b and c), scale bar  = 0.5 mm. *Mean value significantly different from that of control for same time point, *P*≤0.05. 7 d, n = 12–14 per group; 15 d, n = 10 per group. Photomicrographs show granulation tissue from the center of the wound and are oriented such that the epidermis would be on top of the image and the wound margins would be on either side; however, the epidermis and wound margins are not visible at this magnification (20×).

**Figure 4:**
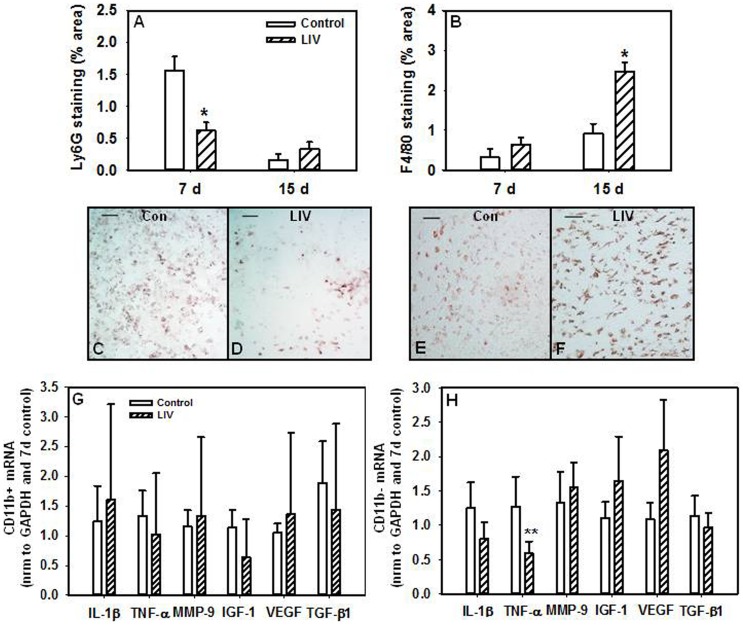
Low-intensity vibration alters inflammatory cell accumulation. Wound sections were stained with antibodies against Ly6C (neutrophils), 7 d, n = 4–10 per group; 15 d, n = 10 per group (a) and F4/80 (macrophages), 7 d, n = 12–14 per group; 15 d, n = 8–9 per group (b). Representative images of Ly6G (c and d) and F4/80 (e and f) stained sections, scale bar  = 0.5 mm. mRNA expression of pro-inflammatory markers IL-1β, TNFα, MMP-9 and pro-healing markers VEGF, IGF-1 and TGF-β1 in CD11b+ (g) and CD11b− (h) cells isolated from wounds by real-time PCR. Data were normalized to GAPDH expression and then to control values, n = 6 per group. *Mean value significantly different from that of control for same time point, *P*≤0.05. **LIV vs. control, *P* = 0.135. Photomicrographs show granulation tissue from the center of the wound and are oriented such that the epidermis would be on top of the image and the wound margins would be on either side; however, the epidermis and wound margins are not visible at this magnification (20×).

In chronic wounds associated with diabetes, reduced levels of pro-angiogenic growth factors contribute to impaired angiogenesis [2]. Importantly, LIV induced a robust increase in angiogenesis on day 7 as assessed by CD31 staining compared to control mice (Figure 3a–c). Furthermore, neutrophil accumulation, which is prolonged in diabetic wounds and contributes to impaired inflammatory resolution [2], was reduced on day 7 (Figure 4a, c, d), while macrophages were increased on day 15 in wounds from LIV-treated versus control mice (Figure 4b, e, f).

### Wound cell phenotype following LIV

We have previously shown that macrophages isolated from wounds of diabetic mice exhibit a sustained pro-inflammatory phenotype and an impaired switch to a pro-healing phenotype [18], [20]. Therefore, we examined whether or not LIV could alter macrophage phenotype in wounds at 7 d post-wounding. LIV did not appear to alter the mRNA expression of pro-inflammatory markers (IL-1β, TNFα, MMP-9) or pro-healing markers (VEGF, IGF-1, TGF-β1) in CD11b+ macrophages compared to controls (Figure 4g). However, a trend towards a less inflammatory cell phenotype was seen in the remaining CD11b− cells from the wound with LIV treatments versus control mice especially as indicated by decreased TNFα expression (Figure 4h), although this did not reach statistical significance (P>0.05).

### LIV increases pro-angiogenic growth factors and chemokines in the wound

**Figure 5:**
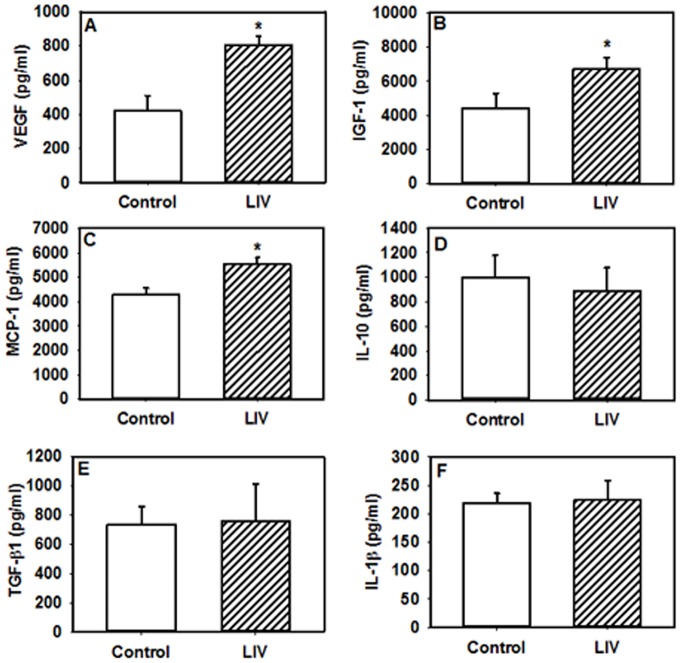
Low-intensity vibration increases levels of pro-angiogenic growth factors in wounds. Mice received either low-intensity vibration (LIV; 0.4 *g* at 45 Hz) or a sham control treatment starting the day of wounding for 5 d/wk. Protein levels of VEGF (a), IGF-1 (b), MCP-1 (c), IL-10 (d), TGF-β1 (e), and IL-1β (f) measured in day 7 wound homogenates using ELISA. *LIV vs. control, *P≤*0.05. n = 8–12 per group.

We and others have previously shown that the impaired wound healing in db/db mice is associated with a sustained upregulation of pro-inflammatory cytokines and down regulation of anti-inflammatory cytokines in wounds compared to non-diabetic mice [18], [21], [22]. To provide insight into whether LIV can promote healing by altering the pro-inflammatory environment of db/db wounds, levels of cytokines and chemokines known to regulate tissue healing were analyzed in wound homogenates at day 7 post-wounding via ELISA. Levels of the pro-inflammatory cytokine IL-1β and the anti-inflammatory cytokine IL-10 were not different between LIV-treated mice and non-vibrated controls (Figure 5d, f); however, LIV led to higher levels of growth factors IGF-1 and VEGF but not TGF-β1 compared to control mice (Figure 5a, b, e). Similarly, levels of the chemokine MCP-1 were higher in LIV-treated mice vs. controls (Figure 5c), which correlates with the higher levels of macrophages in wounds of LIV-treated mice.

## Discussion

Despite the high prevalence and socioeconomic impact of chronic wounds associated with diabetes, effective treatments remain elusive. In this study, we explored a novel therapeutic approach to wound healing using whole-body low-intensity vibration in diabetic mice, which also demonstrate impaired healing. The major finding of this study is that LIV improves wound healing in part by promoting a pro-angiogenic wound environment. Compared to non-vibrated control mice, LIV treatment increased granulation tissue formation and angiogenesis, and accelerated closure and re-epithelialization. These LIV-induced improvements were associated with higher levels of growth factors IGF-1 and VEGF and the chemokine MCP-1 in the wound environment.

As opposed to local mechanical stimulation of the wounds (i.e. negative pressure wound therapy, ultrasound), LIV applies a systemic mechanical stimulus at a low intensity. While the mechanotransduction pathways that modulate the cellular response to LIV remain to be elucidated, it is well documented that LIV can be anabolic to bone and that the mechanical signals do not need to be of large magnitude to elicit an anabolic effect [9], [10], [11], [12]. Using diabetic mice as a model of impaired wound healing, we demonstrate that LIV treatments are able to exert an anabolic effect on cutaneous wounds leading to accelerated healing. These low-level mechanical signals may exert local effects by directly stimulating the production of growth factors, such as IGF-1 and VEGF by various cells in the wound. In addition to local effects, we speculate that LIV may also exert systemic effects, which will be a focus of future investigation. Elucidating the mechanisms underlying the local and/or systemic effects of LIV warrants further investigation.

Diminished production of pro-angiogenic growth factors, such as IGF-1 and VEGF, is thought to contribute to the impaired angiogenesis observed in chronic wounds associated with diabetes [2]. Our data demonstrate that LIV can enhance angiogenesis in diabetic mice as demonstrated by an increase in CD31 staining on day 7. LIV may exert these pro-angiogenic effects via the actions of IGF-1 and VEGF as these levels increased in wound tissue on day 7 with LIV. VEGF can induce angiogenesis by increasing endothelial cell migration and proliferation [23], [24]. IGF-1 has been shown to induce endothelial cell migration via chemotactic activity in endothelial cell lines [25]. Macrophages are also widely associated with angiogenesis and healing [19], [26]; these cells were increased by LIV at day 15 while MCP-1, a key chemokine for monocyte/macrophage migration, increased at day 7. Reasons for this discrepancy are currently unclear. Nonetheless, since the peak in macrophages occurred after granulation tissue formation and angiogenesis peaked, their function during this period may be more related to wound remodeling.

Interestingly, a prolonged expression of MCP-1 in wound tissue has been previously observed in db/db mice and is thought to be responsible for the prolonged neutrophil and macrophage accumulation during the later phases of wound healing, thus contributing to sustained inflammation and impaired closure [27]. However, MCP-1 can also exert pro-angiogenic effects. In hindlimb ischemia, treatment with exogenous MCP-1 was shown to increase monocyte/macrophage recruitment, collateral vessel formation, and blood flow [28], [29]. Furthermore, MCP-1 can directly act on endothelial cells to induce angiogenesis via induction of VEGF-A gene expression and subsequent pro-angiogenic actions of VEGF [30]. In our study, the LIV-induced increase in MCP-1 was paralleled by an increase in VEGF protein and angiogenesis in day 7 wounds, consistent with a pro-angiogenic role of MCP-1 in LIV-induced angiogenesis and wound healing.

Cells such as monocytes/macrophages, fibroblasts and/or endothelial cells promote wound healing in part by producing growth factors that induce angiogenesis, collagen deposition and wound closure [2], [19], [31], [32], [33]. We have previously shown that macrophages isolated from wounds of diabetic mice exhibit a sustained pro-inflammatory phenotype and an impaired switch to a pro-healing phenotype [18], [20]. Therefore, we examined whether or not LIV could alter the phenotype of CD11b+ macrophages or CD11b− cells, which are mainly comprised of fibroblasts, endothelial cells and keratinocytes, isolated from wounds at 7 d post-wounding. Our finding that LIV resulted in a trend towards a less inflammatory phenotype in CD11b− cells, but not in CD11b+ macrophages in the wound suggests LIV may exert pro-angiogenic effects via cells such as fibroblasts, endothelial cells and/or keratinocytes.

The diabetic wound environment can be highly proteolytic with increased expression of proteolytic enzymes, such as MMPs, and a downregulation of their inhibitors, tissue inhibitors of metalloproteinases (TIMPs) and other growth factors. This highly proteolytic environment leads to a diminished extracellular matrix synthesis and increased degradation [34], [35]. In the present study, LIV was able to increase granulation tissue formation on day 7 compared to non-vibrated control mice, which was associated with increases in growth factors IGF-1 and MCP-1, both of which have been shown to regulate the expression of MMPs and or TIMPs, [36], [37]. Furthermore, granulation tissue levels in LIV-treated mice were returned to levels of the control mice by day 15, which may be favorable in that prolonged and excess production of the extracellular matrix can lead to excess scar formation [38].

Given the anabolic and pro-angiogenic effects of LIV on wound healing, LIV treatments may serve as an exercise mimetic for patients experiencing chronic wounds with limited mobility. Exercise training has been shown to improve wound healing in healthy older adults [39] and aged mice [40] as well as in high fat diet-induced obese mice [41]. Interestingly, the improved wound closure in high fat diet-induced obese mice appeared to be independent of changes in inflammatory cytokines and insulin sensitivity [41]. While angiogenesis was not assessed in these studies, a single bout of low-intensity cycling was shown to improve cutaneous perfusion in diabetic patients with peripheral arterial occlusive disease [42], suggesting that exercise training may improve wound healing via pro-angiogenic mechanisms. In support, acute exercise in humans increased numbers of bone marrow-derived endothelial progenitor cells (EPC) in peripheral blood [43], [44], [45], while chronic exercise in wild-type mice expanded both the bone marrow and peripheral blood EPC pools [46]. Importantly, the exercise-induced increase in EPCs was paralleled by an increase in circulating VEGF protein levels [43], [44], [46]. The current study is limited in that the effect of LIV on the mobilization of bone marrow-derived cells was not assessed and mechanistic evidence to support the pro-angiogenic effects of LIV are still needed. Nonetheless, the LIV-induced improvements in wound healing coupled with the increase in angiogenesis and VEGF in the wound suggests LIV may exert pro-angiogenic effects similar to exercise training.

Other limitations of this study include the fact that type 2 diabetes in db/db mice is induced by mutation of a single gene, whereas in humans, the disease is polygenic. In addition, in db/db mice, healing is delayed but wounds do not become truly chronic. However, there are similarities between the healing responses in db/db mice and diabetic humans, including prolonged accumulation of monocytes/macrophages and pro-inflammatory cytokines and proteases, reduced levels of various growth factors, delayed closure, and reduced angiogenesis and matrix deposition [2], [21], [22], [27], [47], [48], [49], [50], [51].

In summary, LIV may provide a novel therapeutic avenue for promoting angiogenesis and healing of diabetic wounds. Future studies that optimize the LIV protocols to maximize pro-healing effects and further elucidate the mechanisms through which LIV exerts local and/or systemic effects to improve wound healing are warranted. Importantly, the LIV protocol can feasibly be translated to clinical trials for diabetic patients with chronic wounds since the equipment utilized has already been used to ameliorate bone loss in young and elderly human subjects [9], [10], [11], [12].
